# Mutation of Phe318 within the NPxxY(x)_5,6_F motif in melanin-concentrating hormone receptor 1 results in an efficient signaling activity

**DOI:** 10.3389/fendo.2012.00147

**Published:** 2012-11-26

**Authors:** Akie Hamamoto, Manabu Horikawa, Tomoko Saho, Yumiko Saito

**Affiliations:** ^1^Graduate School of Integrated Arts and Sciences, Hiroshima UniversityHiroshima, Japan; ^2^Bioorganic Research Institute, Suntory Foundation for Life SciencesOsaka, Japan

**Keywords:** GPCR, helix 8, melanin-concentrating hormone, NPxxY(x)_5,6_F motif, signal transduction

## Abstract

Melanin-concentrating hormone receptor 1 (MCHR1) is a G-protein-coupled receptor (GPCR) that plays an important role in feeding by coupling to Gα_q_- and Gα_i_-mediated signal transduction pathways. To interrogate the molecular basis for MCHR1 activation, we analyzed the effect of a series of site-directed mutations on rat MCHR1 function. In the highly conserved NPxxY(x)_5,6_F domain of GPCRs, the phenylalanine residue is involved in structural constraints; replacement with alanine generally leads to impaired/lost GPCR function. However, Phe-to-Ala (F318A) mutation in MCHR1 had no significant effect on the level of cell surface expression and receptor signaling. By analyzing a further series of mutants, we found that Phe-to-Lys substitution (F318K) caused the most significant reduction in the EC_50_ value of MCH for calcium mobilization without affecting receptor expression at the cell surface. Interestingly, GTPγS-binding, which monitors Gα_i_ activation, was not modulated by F318K. Our results, combined with computer modeling, provide new insight into the role of Phe in the NPxxY(x)_5,6_F motif as a structurally critical site for receptor dynamics and a determinant of Gα protein interaction.

## Introduction

Mammalian melanin-concentrating hormone (MCH), a cyclic nonadecapeptide produced predominantly by neurons of the lateral hypothalamus, is involved in the regulation of food intake behavior and energy expenditure (Bittencourt et al., 1992; Rossi et al., 1997; Shimada et al., 1998). MCH acts *via* two G-protein-coupled receptors (GPCRs), Melanin-concentrating hormone receptor 1 (MCHR1), and MCHR2 (Chambers et al., 1999; Saito et al., 1999; An et al., 2001), of which MCHR2 is not functionally present in rodents (Tan et al., 2002). MCHR1 is widely expressed at high levels in the brain (Saito et al., 2001). Because mice lacking MCHR1 are lean, hyperactive, hyperphagic, and hypermetabolic (Chen et al., 2002; Marsh et al., 2002), MCHR1 is viewed as the physiologically relevant MCH receptor in rodents. In support of this belief, selective MCHR1 antagonists decrease food intake and body weight in both normal and diet-induced obese rats (Takekawa et al., 2002; Shearman et al., 2003). Moreover, some of these antagonists exhibit anti-depressant and anxiolytic effects (Borowsky et al., 2002; Georgescu et al., 2005). Therefore, the MCH-MCHR1 system could be an important target for the treatment of obesity and certain mood disorders.

In mammalian cells transfected with MCHR1, MCH is able to activate multiple signaling pathways including calcium mobilization, activation of extracellular signal-regulated kinase (ERK) and inhibition of cyclic AMP generation through Gα_i/o_- and Gα_q_-coupled pathways (Chambers et al., 1999; Saito et al., 1999; Hawes et al., 2000). Several studies have reported structural determinants of MCHR1 activation by MCH. Biochemical analysis of MCHR1 using molecular modeling identified Asp123 in the third transmembrane domain (TM3) as being crucial for ligand binding (Macdonald et al., 2000). In addition, Thr255, which is located at the junction of intracellular loop 3 (i3) and transmembrane domain 6 (TM6), is critically important for receptor folding and correct trafficking to the cell surface (Fan et al., 2005). We previously identified that Asn23 in the extracellular N-terminus contributed mainly to N-linked glycosylation of MCHR1 and is necessary for MCHR1 cell surface expression, ligand binding and signal transduction (Saito et al., 2003). We also showed that Arg155 in intracellular loop 2 (i2) and a proximal dibasic motif (Arg319/Lys320) in eighth cytoplasmic helix (helix 8: a common short amphiphilic helical domain in the proximal C-terminal tail) are important for signaling (Tetsuka et al., 2004; Saito et al., 2005), whereas the distal part of the C-terminal tail is necessary for receptor internalization (Saito et al., 2004). However, despite numerous mutagenesis studies, the residues that determine G protein selectivity (Gα_q_ vs. Gα_i_) have yet to be identified.

The NPxxY(x)_5,6_F sequence, located at the junction between TM7 and the connecting cytosolic helix 8, is conserved in most rhodopsin family (class A) GPCRs, including the MCH receptor (Gether, 2000; Huynh et al., 2009). The high degree of conservation of this motif suggests that it must play very important roles in rhodopsin family GPCR functionality. Mutations in the NPxxY(x)_5,6_F motif are reported to affect ligand binding, G protein coupling and receptor phosphorylation. In rhodopsin, the prototypical GPCR, the Tyr and Phe residues within the motif were both found to be critical for proper light-induced conformational changes from the ground state (Acharya and Karnik, 1996). Moreover, the Phe residue is reported to be essential for export of the β1-adrenergic receptor (β1-AR), α2B-adrenergic receptor (α2B-AR) and A1 adenosine receptor from the endoplasmic reticulum (ER) (Delos Santos et al., 2006; Duvernay et al., 2009; Málaga-Diéguez et al., 2010). Indeed, Phe-to-Ala substitution in the α2B-AR dramatically reduced cell-surface expression by 91% compared with their wild-type variants (Duvernay et al., 2009). To determine the role of the conserved Phe residue (F318) in the NPxxY(x)_5,6_F motif present in the MCHR1, we examined the effect of site-directed mutagenesis of this residue on receptor function, and noted a most significant increase in calcium mobilization relative to wild-type after substitution of F318 with a positively-charged lysine residue. Our analyses show that Lys replacement mutation (F318K) produces an efficient signaling property that selectively increases Gα_q_-mediated pathway without changing cell surface expression. We further discuss the significance of the position of Phe318 using a homology docking model of MCHR1 with Gα_q_ and Gα_i_ proteins, respectively. To date, this is the first study to provide meaningful insights into the relationship between conformational changes in MCHR1 and G protein activation.

## Materials and methods

### cDNA constructs for MCHR1 and mutant receptors

The generation of a cDNA encoding a Flag epitope tag before the first methionine in rat MCHR1 (NM_031758/GenBank/EMBL) was described previously (Saito et al., 2003). Single-substitution mutations of the NPxxY(x)_5,6_F domain were produced by oligonucleotide-mediated site-directed mutagenesis using a QuikChange site-directed mutagenesis kit (Stratagene, La Jolla, CA, USA). All mutations in the MCHR1 cDNA sequence were confirmed by sequencing analysis. Mutated MCHR1 cDNAs were excised by digestion with EcoRI and XhoI and inserted into the pcDNA3.1 expression vector.

### Cell culture and transient transfection

HEK293T cells were cultured in DMEM containing 10% fetal bovine serum. The plasmid DNA was mixed with LipofectAMINE PLUS transfection reagent (Life Technologies Corporation, Carlsbad, CA, USA) and the mixture was diluted with OptiMEM and added to the cells (Saito et al., 2003). For western blotting and GTPγS-binding assays, the cells were re-seeded onto 6-well plates. Cells were re-plated onto LAB-TEK 8-well plates (Nunc, Rochester, NY, USA) for immunocytochemistry, and onto 24- and 96-well plates (BIOCOAT, Becton Dickinson, Belford, MA, USA) for FACScan flow cytometric analysis and the calcium influx assay, respectively. The re-plated cells were cultured for a further 18–24 h.

### Western blotting for MCHR1

To generate whole cell extracts, transiently-transfected HEK293T cells were lysed with ice-cold sodium dodecyl sulfate sample buffer [50 mM Tris-HCl (pH 6.8), 2% sodium dodecyl sulfate, 50 mM β-mercaptoethanol, and 10% glycerol], then homogenized at 4°C by sonication (SONICAOR Ultrasonic processor W-225, Wakenyaku Ltd., Kyoto, Japan) using 5×30 s bursts at 20% power. Aliquots containing 15 μg of total protein were separated by SDS-PAGE and electro-transferred to Hybond-P PVDF membranes (GE Healthcare UK Ltd., Little Chalfont, UK). After blocking with 5% skim milk, membrane-expressed Flag-MCHR1 was detected using 0.5 μg/ml anti-Flag M2 antibody (Wako, Osaka, Japan), followed by a horseradish peroxidase-conjugated goat anti-mouse IgG antibody (Saito et al., 2005). Reactive bands were visualized with enhanced chemiluminescence (ECL) reagent (GE Healthcare UK Ltd.).

### FACScan flow cytometric analysis of cell surface receptors

Transfected HEK293T cells in 24-well plates were fixed with 1.5% paraformaldehyde-PBS solution for 10 min at room temperature, then incubated with 0.25 μg/ml anti-Flag M2 antibody in PBS containing 20% FBS for 1 h. The cells were washed three times with PBS and then incubated with Alexa Fluor 488-conjugated goat anti-mouse IgG secondary antibody (Molecular Probes, Eugene, OR, USA) for 1 h (Tetsuka et al., 2004; Saito et al., 2005). The cells were washed, harvested with 5 mM EDTA and analyzed using a FACSCalibur flow cytometer (BD, Franklin Lakes, NJ). Cells were gated by light scatter or exclusion of propidium iodide, and 10,000 cells were acquired for each time point. The mean fluorescence of all cells minus the mean cell fluorescence with the Alexa Fluor 488-conjugated secondary antibody only was used for the calculations.

### Immunofluorescence microscopy

Transfected HEK293T cells were fixed in a 3.7% paraformaldehyde-PBS solution for 10 min. After two washes with PBS, the cells were transferred, either with or without permeabilization using 0.05% Triton X-100 in PBS for 15 min, into a blocking solution (20% goat serum in PBS) for 30 min, then incubated with 0.5 μg/ml anti-Flag M2 antibody for 1 h. The anti-Flag M2 antibody was detected using Alexa Fluor 488-conjugated goat anti-mouse IgG secondary antibody. Fluorescence imaging was performed using a BZ-9000 microscope (Keyence, Tokyo, Japan). For fluorescence imaging of MCH-induced receptor internalization, cells were pre-incubated at 37°C in serum-free DMEM for 3 h. Cells were then incubated with 1 μM rat MCH for 10, 30, and 60 min at 37°C in a 5% CO_2_ incubator. Cells were fixed, permeabilized and then incubated with 0.5 μg/ml anti-Flag M2 antibody in PBS containing 20% FBS for 1 h. The cells were washed three times with PBS and then incubated with Alexa Fluor 488-conjugated goat anti-mouse IgG secondary antibody for 1 h. Fluorescence imaging was performed using a FLUOVIEW FV1000 confocal microscope (Olympus, Tokyo, Japan).

### Measurement of intracellular CA^2+^

Measurement of intracellular Ca^2+^ was performed as described previously (Saito et al., 2003, 2005; Tetsuka et al., 2004). Transiently transfected cells seeded in 96-well plates were loaded with a non-wash calcium dye (Calcium Assay Kit 5, Molecular Devices Japan, Tokyo, Japan) in Hank's balanced salt solution containing HEPES (pH 7.5) for 1 h at 37°C. For each concentration of MCH, the level of [Ca^2+^]_i_ was detected using a FlexStation 3 Microplate Reader (Molecular Devices). The data were expressed as fluorescence (arbitrary units) vs. time. The EC_50_ values for MCH were obtained from sigmoidal fits using a non-linear curve-fitting program (Prism v3.0; GraphPad Software, San Diego, CA, USA). Rat/mouse/human MCH and Compound 15 were purchased from Peptide Institute (Osaka, Japan) and Bachem AG (Bubendorf, Switzerland), respectively.

### GTPγS-binding assay

GTPγS-binding assay was performed as described previously (Saito et al., 2005). Aliquots (10 μ g) of membrane proteins were incubated in GTPγS binding buffer (20 mM HEPES-NaOH pH 7.5, 100 mM NaCl, 5 mM MgCl_2_, 0.2% BSA and 3 μ M GDP) containing 0.2 nM [^35^S]GTPγS (PerkinElmer, Santa Clara, CA, USA) and various concentrations of MCH for 30 min at 30°C. To determine the non-specific binding, unlabeled GTPγS was added to the binding mixtures to a final concentration of 100 μ M. Bound [^35^S]GTPγS was separated from free [^35^S]GTPγS by rapid filtration through GF/C filters and washed with ice-cold binding buffer. Filters were then immersed in scintillation cocktail (Emulsion-Scintillator Plus; Packard Bioscience, Groningen, The Netherlands) and trapped radioactivity counted using a liquid scintillation counter.

### Molecular modeling

To generate homology models of rat MCHR1 mutant F318K, we used the X-ray structure of constitutively active rhodopsin bound to the C-terminus peptide of the α-subunit of the G protein, transducin (PDB code 2X72), as a structural template. Alignment analysis in sequences of rat MCHR1 and rhodopsin was performed using CLUSTALW2.0 installed in Genetyx v9.0 (Genetyx Corporation, Tokyo, Japan). Initial models of rat MCHR1 mutant F318K with K341L transducin C-terminus peptide (340–350; ILENLKDCGLF) was constructed using the Modelor module installed in Discovery Studio (DS) v3.0 (Accelrys, Tokyo, Japan). After replacement of L341 and E342 to Lys and Asn, the structure of rat MCHR1(F318K) bound to a C-terminus peptide (343–353; IKNNLKDCGLF) of the Gα_i_ subunit was optimized using the molecular mechanics and molecular dynamics simulation with a CHARMm force field in the DS. Furthermore, replacement of the Gα_i_ peptide with a C-terminus peptide to the Gα_q_ subunit (349–359; LQLNLKEYNLV) and subsequent similar optimization in DS provided the model structure of rat MCHR1(F318K) bound to the Gα_q_ C-terminus peptide. The X-ray structure of rhodopsin (PDB code 2X72) and the two constructed models (rat MCHR1(F318K)-Gα_i_ peptide and rat MCHR1(F318K)-Gα_q_ peptide) were superimposed for comparison.

## Results

### Effects of various single-site Ala substitution mutations of the conserved NPxxY(x)_5,6_F motif on receptor expression and activity

**Figure 1:**
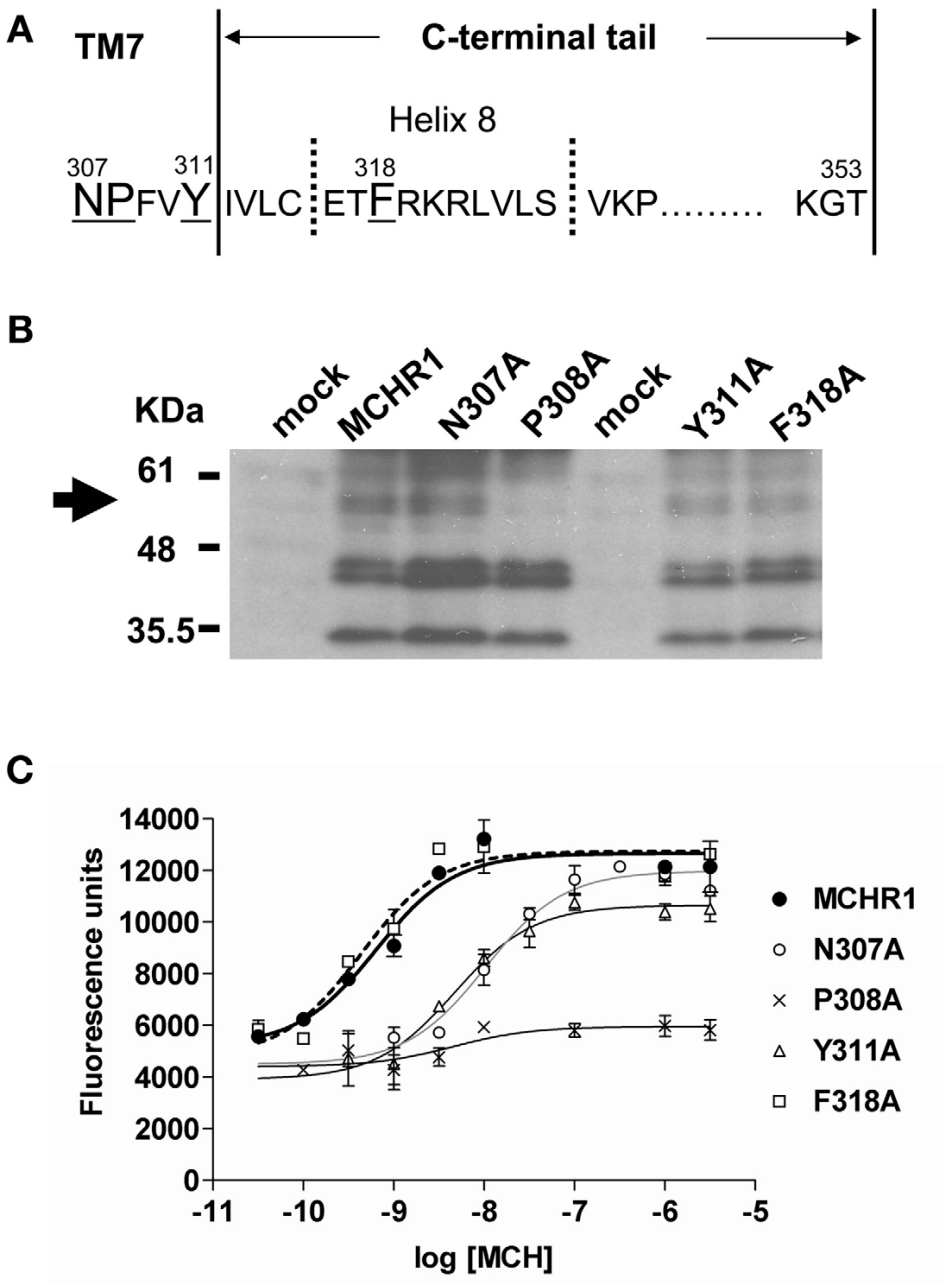
**Analysis of the effects of individual substitution mutations in the NPxxY(x)_5,6_F motif of MCHR1 on receptor function in HEK293T cells. (A)** Schematic representation of the conserved NPxxY(x)_5,6_F motif and the C-terminal tail of the rat MCHR1. The C-terminal tail of rat MCHR1 extends 42 residues from the plasma membrane (residue 312–353) and is predicted to include an eighth cytoplasmic helix (helix 8) (Tetsuka et al., 2004). Amino acid residues targeted for mutational analyses are underlined. TM7; transmembrane 7. **(B)** Protein expression of Flag-MCHR1 and mutant receptors. After lysis of transfected cells with SDS-sample buffer, 15 μ g total protein was separated by 15% SDS-PAGE, transferred to a polyvinyl difluoride membrane, and immunoblotted with an anti-Flag M2 antibody. Four major immunoreactive bands of 35, 44, 45, and 60 kDa are present in Flag-MCHR1 and individual mutant receptors. **(C)** Cells transfected with Flag-MCHR1 or substitution mutant receptors were stimulated with the indicated concentrations of MCH, and the subsequent changes in cytoplasmic free Ca^2+^ levels were measured using a FlexStation. Results shown are representative of at least three-independent experiments.

First, to analyze the function of the NPxxY(x)_5,6_F motif, a series of Ala-substituted mutants were generated, as shown in Figure 1A. We transiently transfected Flag-tagged MCHR1 or mutant receptors into HEK293T cells, then examined receptor expression levels by western blotting analysis using an anti-Flag M2 antibody. Several immunoreactive bands were detected in the whole lysate isolated from cells expressing Flag-MCHR1 (Figure 1B), some of which corresponded to the predicted molecular masses of MCHR1 variants (approximately 35, 44, 45, and 60 kDa (Saito et al., 2003, 2005; Tetsuka et al., 2004), although additional immunoreactive bands were observed at 45–60 kDa. Our previous study revealed that the 35-kDa band is the non-glycosylated form of MCHR1 (Saito et al., 2003), while the three higher molecular mass bands are different *N*-linked glycosylated forms. The migration patterns of N307A, Y311A, and F318A were very similar and no significant reductions in the intensity of the higher molecular mass bands were observed relative to Flag-MCHR1. However, the pattern in P308A was different, with the expression of the higher molecular mass band at 60 kDa apparently drastically reduced (Figure 1B, arrow). This phenomenon is likely caused by a lack of appropriate glycosylation of the mutant receptors, as previously shown in an-*N*-linked glycosylation study and other MCHR1 studies (Saito et al., 2003; Aizaki et al., 2009). The cell surface expression levels of Flag-MCHR1 and mutants containing Ala-substitutions in the NPxxY(x)_5,6_F motif were monitored by FACScan flow cytometry using an anti-Flag M2 antibody. Transient transfection of N307A and Y311A gave expression levels of 23% and 30%, respectively, relative to that of Flag-MCHR1, while the F318A mutation was expressed at approximately the same level as the non-mutated control (Table 1). Conversely, P308A expression was reduced by more than 90% compared to Flag-MCHR1, suggesting that the mutant was mostly retained intracellularly (Figure 1B and Table 1).

**Table 1:** **Cell surface expression of Flag-MCHR1 and variants transiently expressed in HEK293T cells**.

**Receptor**	**Cell surface expression (FACS, %)**
Flag-MCHR1	100
N307A	77.3 ± 0.3*^b^*
P308A	6.3 ± 1.0*^b^*
Y311A	69.3 ± 9.2*^a^*
F318A	91.0 ± 8.7
F318R	90.5 ± 9.5
F318K	96.2 ± 5.8
F318P	90.2 ± 9.1

The data represent the mean ± S.E.M of three or four-independent experiments performed in triplicates.

a P < 0.05, significantly different from Flag-MCHR1 by Student's t-test.

b P < 0.01, significantly different from Flag-MCHR1 by Student's t-test.

Next, we assessed the capacity of receptors containing alanine mutations in their NPxxY(x)_5,6_F motif to induce intracellular signals in response to MCH. MCH-induced calcium influx was quantified in transiently transfected cells using a FlexStation 3 Microplate Reader. Mock-transfected HEK293T cells acted as a negative control and did not respond to MCH stimulation (data not shown). Considerable evidence suggests that most single-substitution mutations of highly conserved amino acids (such as the DRY or NPxxY(x)_5,6_F motifs) lead to impairment or inactivation of receptor protein signaling. Indeed, substitution of conserved Pro308 with Ala (P308A) resulted in a dramatic attenuation of cell surface expression (Table 1). Therefore, this receptor did not respond to MCH by calcium mobilization (Figure 1C, Table 2), even when challenged with a high concentration (10 μ M) of MCH.

**Table 2:** **Calcium mobilization stimulated by MCH via Flag-MCHR1 and variants [containing various single point mutations in the highly conserved NPxxY(x)_5,6_F motif] expressed in HEK293T cells**.

**Receptor**	**EC_50_ of MCH (nM)**	**Maximum response (%)**
Flag-MCHR1	1.5 ± 0.4	100
N307A	32.5 ± 9.0	83.5 ± 5.4
P308A	–	–
Y311A	12.2 ± 2.3	77.3 ± 10.0
F318A	1.2 ± 0.3	101.0 ± 8.2

P308A does not respond to MCH (–). The results represent the mean ± S.E.M. of at least three-independent experiments performed in duplicate.

Substitution of either Asn307 or Tyr311 with Ala also significantly affected MCH-induced calcium mobilization. N307A and Y311A mutant receptors exhibited a maximal response that was 20–30% lower than wild-type receptors and had EC_50_ values that were 21- and 8-fold higher, respectively, than Flag-MCHR1 (Table 2). However, the effects of alanine mutation of Phe318 in the highly conserved motif were distinct from other mutants (Delos Santos et al., 2006; Duvernay et al., 2009; Málaga-Diéguez et al., 2010; Kaye et al., 2011). The EC_50_ and maximal response of MCH-induced calcium signaling in cells expressing the F318A mutant were essentially identical to those of Flag-MCHR1. This is consistent with our previous data (Tetsuka et al., 2004). These results indicate that the conserved Phe in MCHR1 has a distinct signaling role as compared with other conserved amino acids in the NPxxY(x)_5,6_F motif.

### Effects of individual substitution of highly conserved Phe on MCHR1 function

**Figure 2:**
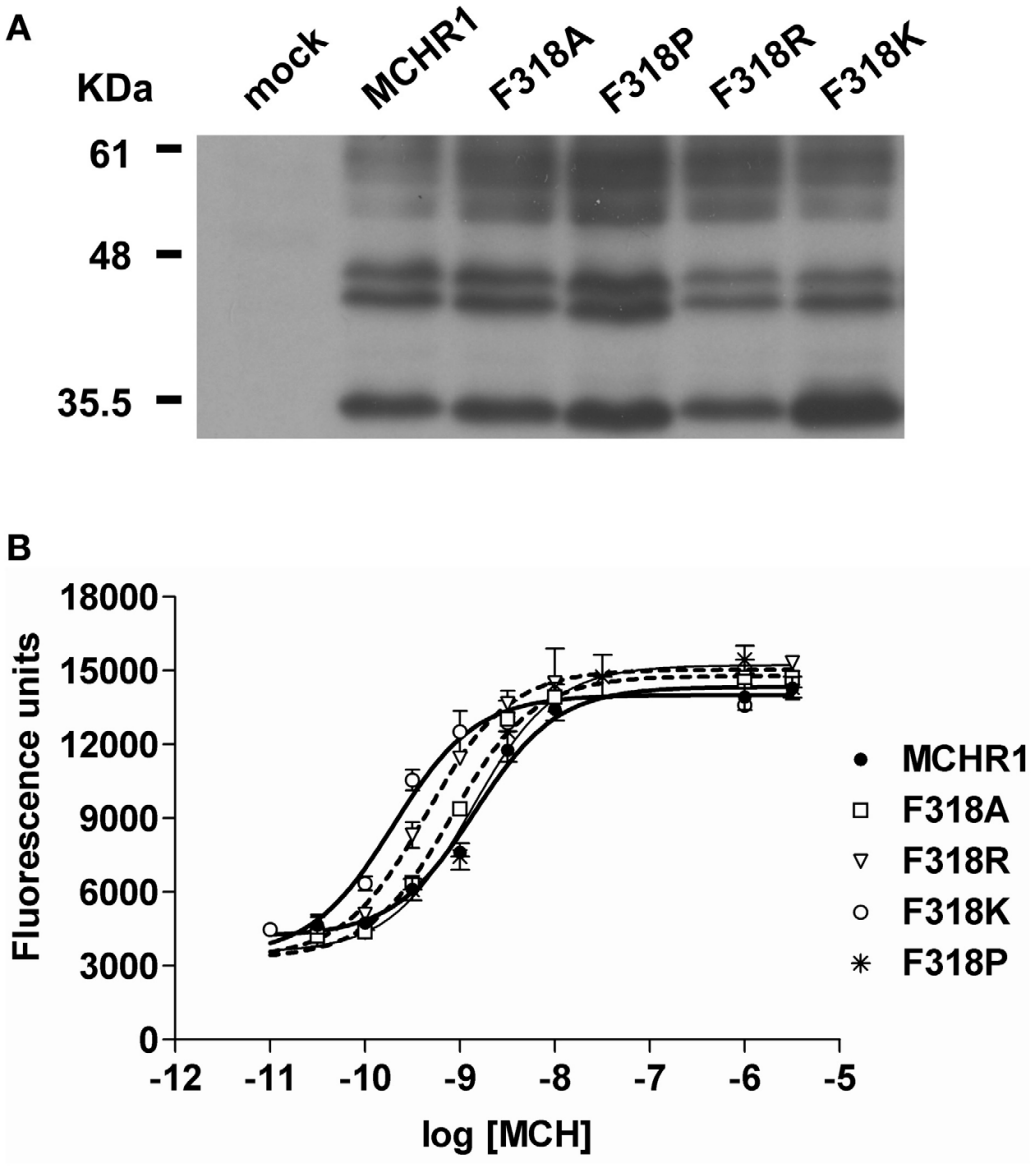
**Analysis of the effects of substitution mutations of the highly conserved Phe318 in the MCHR1 NPxxY(x)_5,6_F motif on receptor function in HEK293T cells. (A)** Protein expression of Flag-MCHR1 and mutant receptors. After lysis of transfected cells with SDS-sample buffer, 15 μ g total protein were separated by 15% SDS-PAGE, transferred to a polyvinyl difluoride membrane, and immunoblotted with an anti-Flag M2 antibody. Four major immunoreactive bands of 35, 44, 45, and 60 kDa are present in Flag-MCHR1 and individual mutant receptors. **(B)** Dose-response relationship of MCH-stimulated calcium influx in HEK293T cells expressing Flag-MCHR1 or mutant receptors. Cells transfected with Flag-MCHR1 or the substitution mutant receptors were stimulated with the indicated concentrations of MCH, and the subsequent changes in cytoplasmic free Ca^2+^ levels were measured using a FlexStation. Results shown are representative of at least three-independent experiments.

**Figure 3:**
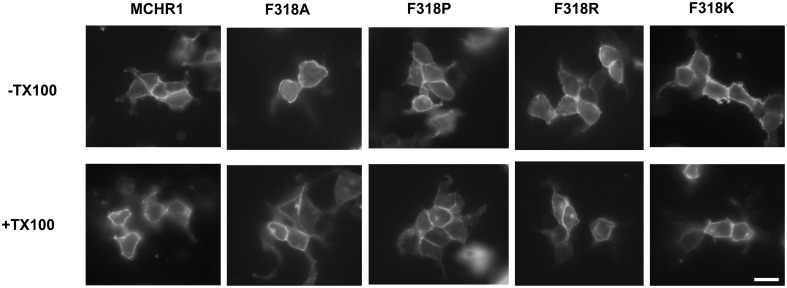
**Confocal immunolocalization of Flag-MCHR1 and the mutant receptors using an anti-Flag M2 antibody in HEK293T cells.** Cell surface expression was compared using transfected non-permeabilized cells (-TX100, without Triton X-100; upper row) and permeabilized cells (+TX100, with Triton X-100; lower row). Vector-transfected cells incubated with the anti-Flag M2 antibody showed no significant staining (data not shown). Bar, 10 μm.

**Figure 4:**
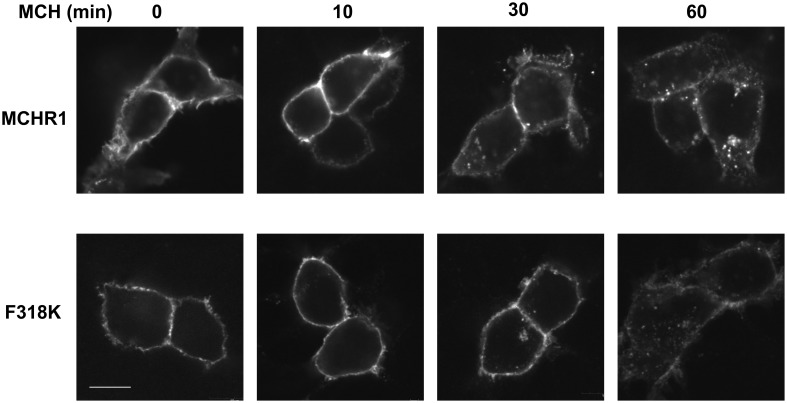
**MCH-mediated receptor internalization in HEK293T cells.** Cells expressing MCHR1 or the F318K mutant were stimulated with 1 μ M MCH for the time shown, fixed and imaged by confocal fluorescence microscopy. *Prior* to MCH addition, cells were incubated with serum-free DMEM for 3 h. Bar, 10 μm.

To further analyze the effect of the Phe318 substitution in the NPxxY(x)_5,6_F motif, we performed site-directed mutagenesis of Phe318 to proline, a change that is thought to disrupt helix formation and may cause drastic changes in receptor function. We also mutated Phe to Arg and Lys, because these substitutions impart a positive charge to the position. Analysis of receptor expression levels by western blotting analysis with the anti-Flag M2 antibody showed that the migration patterns of F318A, F318P, F318R, and F318K were very similar and no drastic reduction in the intensity of the higher molecular mass bands were observed compared to Flag-MCHR1 (Figure 2A). We next determined the expression characteristics of each mutant by quantifying cell surface expression and observing subcellular distribution. The cell surface expression levels of F318A, F318P, F318R, and F318K caused no significant decrease as compared to the Flag-MCHR1 (Table 1). Antibody staining in non-permeabilized cells revealed that F318A, F318P, F318R, and F318K were clearly localized in the plasma membrane, and their labeling intensities were approximately equivalent with that of Flag-MCHR1 (Figure 3, upper). In permeabilized cells, all four mutants were also predominantly detected in the plasma membrane, as was Flag-MCHR1 (Figure 3, bottom). These results confirmed that both the level of cell surface expression and the subcellular localization were unaffected by the Phe318 substitution in the NPxxY(x)_5,6_F motif. This is in marked contrast with similar mutants of other GPCRs including the α2B-AR (Delos Santos et al., 2006; Duvernay et al., 2009; Málaga-Diéguez et al., 2010), in which membrane translocation was dramatically impaired by mutation of the conserved Phe residue in the NPxxY(x)_5,6_F motif. Using fluorescence microscopy, we also examined the features of receptor internalization following exposure to MCH, because rat MCHR1 undergoes rapid MCH-induced internalization during the 30 min after stimulation (Saito et al., 2004). Consistent with this previous study, treatment with MCH for 30 min caused a loss of membrane localization and the appearance of MCHR1-containing vesicles. This distribution was also observed following 60 min of incubation. A similar time-course of MCH-induced receptor internalization was obtained for the F318K mutant (Figure 4).

The effects of Phe318 mutations on MCHR1 responsiveness to MCH in calcium mobilization are shown in Table 3 and Figure 2B. In cells expressing single-substitution mutants, the MCH EC_50_ was 7.8 nM and 0.8 nM for F318P and F318R, respectively, with identical maximal responses. Importantly, MCH had increased potency in releasing calcium *via* the F318K mutant receptor (EC_50_ = 0.4 ± 0.1 nM, compared with 2.5 ± 0.8 nM for Flag-MCHR1, Table 3), although the maximal response was unchanged. We also mutated the uncharged hydrophobic Phe to other uncharged polar amino acid residues (Gly, Ser, and Cys), a different hydrophobic residue (Trp) and a different basic residue (His) and measured the responsiveness of the resultant mutant receptors in the calcium mobilization assay (Table 3). Among the mutants, F318K and F318R exhibited significantly enhanced cellular signaling, but F318K caused a higher responsiveness than F318R. In addition to using MCH itself, we also tested the activity of the mammalian MCH analog, Compound 15, which efficiently binds with high affinity to MCHR1 (Bednarek et al., 2002). The potency of Compound 15 was also enhanced in cells expressing F318K compared to Flag-MCHR1 (0.15 ± 0.10 nM vs. 1.3 ± 0.4 nM, respectively; mean ± S.E.M. from three-independent experiments). This enhanced response to MCH was also observed when F318K was transiently transfected into CHO cells and COS7 cells (data not shown).

**Table 3:** **Calcium mobilization by MCH *via* Flag-MCHR1 and variants [containing single point mutations at F318 in the NPxxY(x)_5,6_F motif] expressed in HEK293T cells**.

**Receptor**	**EC_50_ of MCH (nM)**	**Maximum Response (%)**
Flag-MCHR1	2.5 ± 0.8	100
F318A	1.2 ± 0.2	92.4 ± 7.2
F318P	7.8 ± 3.2^*a*^	93.7 ± 11.4
F318R	0.8 ± 0.3^*b*^	99.4 ± 3.0
F318K	0.4 ± 0.1^*b*^	102.0 ± 8.0
F318G	2.3 ± 0.9	99.6 ± 9.8
F318S	2.4 ± 0.7	104.7 ± 18.7
F318C	9.7 ± 1.2^*b*^	101.3 ± 17.7
F318W	3.1 ± 1.3	102.6 ± 9.2
F318H	3.6 ± 0.7	92.3 ± 16.7
E316K	2.2 ± 0.5	99.7 ± 10.9
T317K	3.8 ± 0.9	82.6 ± 6.4^*a*^
R319K/R321K	2.6 ± 0.5	84.8 ± 9.5^*a*^

Results represent the mean ± S.E.M. of at least three-independent experiments performed in duplicate.

a P < 0.05, significantly different from Flag-MCHR1 by Student's t-test.

b P < 0.01, significantly different from Flag-MCHR1 by Student's t-test.

**Figure 5:**
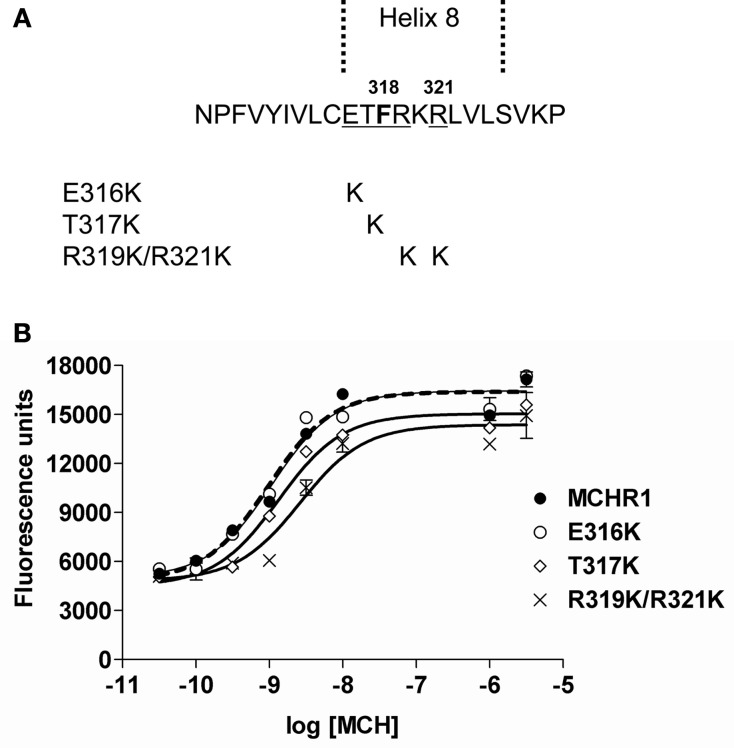
**Effects on receptor activity of substitution mutations of the residues adjacent to Phe318 in HEK293T cells. (A)** Sequences of Flag-MCHR1 and three mutants for which native residues were replaced with Lys (E316K, T317K and R319K/R321K). **(B)** Dose-response relationship of MCH-stimulated calcium influx in HEK293T cells expressing Flag-MCHR1 or substitution mutant receptors. Cells transfected with Flag-MCHR1 or substitution mutant receptors were stimulated with the indicated concentrations of MCH, and the subsequent changes in cytoplasmic free Ca^2+^ levels were measured using a FlexStation. Results shown are representative of at least three-independent experiments.

To elucidate further the effect of Lys mutation, mutagenesis of single or multiple residues around Phe318 was performed, as shown in Figure 5. The E316K, T317K, and R319K/R321K mutations resulted in no higher potency or efficacy of MCH in the calcium mobilization assay. Instead, the T317K and R319K/R321K mutants had a significantly depressed maximal response relative to Flag-MCHR1 (Table 3, Figure 5). Taken together, the activity of MCHR1 in calcium mobilization is enhanced by the introduction of a positively charged Lys residue at the 318 position, but not at the adjacent amino acid residues, indicating that the 318 position seems to be critical for receptor conformation and/or receptor interaction with the Gα proteins that mediate calcium signaling. We then investigated the effects of a similar mutation in human MCHR2, the human ortholog of rat MCHR1. The corresponding Phe in MCHR2 was mutated to Lys (F313K) and tested for MCH responsiveness in calcium mobilization, since MCHR2 is known to couple exclusively to Gα_q_ (An et al., 2001). As shown in Table 4, however, F313K was no more responsive than the non-mutated receptor, and rather showed a 22% reduced maximal response. These results imply that the effect of the Phe-to-Lys substitution on receptor signaling seems to be a specific and intrinsic feature of MCHR1.

**Table 4:** **Signaling of human MCHR2 and its single-substitution (F313K) mutant after transfection into HEK293T cells**.

**Receptor**	**EC_50_ of MCH (nM)**	**Maximum response (%)**
MCHR2	5.2 ± 2.5	100
F313K/MCHR2	5.4 ± 2.4	78.7 ± 7.7

Since the F313 residue corresponds to F318 in rat MCHR1, the F313K mutant of human MCH2R was analyzed for its effect on receptor signaling to calcium mobilization. The data represent the mean ± S.E.M of three-independent experiments performed in duplicate.

### Selectivity of F318K for G proteins in MCHR1

MCHR1-stimulated calcium signaling is mediated through both Gα_q_- and Gα_i/o_-dependent pathways (Hawes et al., 2000; Tetsuka et al., 2004). To confirm which G protein was primarily responsible for the enhancement of calcium signaling by the F318K receptor, we used pertussis-toxin (PTX) to uncouple Gα_i/o_ from MCHR1. In cells expressing Flag-MCHR1, PTX pretreatment increased the EC_50_ value to 3.6 ± 0.6 nM (compared to 1.1 ± 0.3 nM in untreated cells), which equates to a +PTX (PTX-insensitive, Gα_q_-dependent response)/-PTX (combined Gα_i/o_ and Gα_q_-dependent response) ratio of 3.30 (i.e., 3.6/1.1 nM). Following PTX pretreatment, the EC_50_ of MCH in cells expressing F318K was 0.48 ± 0.08 nM (compared to 0.13 ± 0.03 nM in untreated cells), giving a +PTX/−PTX ratio of 3.70. The maximal calcium response to MCH at either receptor was not affected by the addition of PTX. Because PTX reduced the potency of MCH but did not abolish its effect, it is clear that F318K and Flag-MCHR1 both drive calcium influx by a combination of Gα_q_- and Gα_i/o_-dependent pathways. Because MCH still stimulated calcium influx at a lower agonist concentration in F318K after treatment of PTX (EC_50_ = 3.6 nM at Flag-MCHR1 vs. 0.48 nM at F318K), it is clear that PTX-insensitive Gα_q_ protein is involved in mediating calcium responses in both receptors. However, the ratio of MCH potency in the presence and absence of PTX was remarkably similar in both mutated and non-mutated receptors, suggesting that the selectivity of the mutant receptor is likely to be the same as in the wild-type receptor (i.e., that the mutation has not introduced a strong preference for Gα_q_). However, calcium signals by Gα_q_ tend to dwarf those induced by Gα_i/o_ in HEK293T cells (Tetsuka et al., 2004), so it is difficult to judge what effect the mutation has on the ability of the receptor to couple to Gα_i/o_. Thus, it was necessary to analyze other signal transduction events related to MCHR1-Gα_i/o_ protein interactions.

It has been shown previously that MCH stimulation of cells expressing MCHR1 can activate a Gα_i/o_-mediated pathway to cause a decrease in adenylyl cyclase activity, thus reducing cAMP production (Chambers et al., 1999; Saito et al., 1999; Hawes et al., 2000). We therefore tried to measure the interaction between MCHR1 and Gα_i/o_ in cells where cAMP accumulation had been induced by forskolin. Although stable clones for F318K were established, their signaling profiles were very different to transiently transfected cells. For instance, F318K clones were unable to inhibit forskolin-stimulated cAMP accumulation, even at MCH concentrations up to 100 nM MCH (data not shown). These results suggest that the F318K stable clones have lost the ability to couple to Gα_i/o_ protein. Because the amount of total protein and the glycosylation pattern of these proteins in cells were equivalent to that of Flag-MCHR1 (as determined by western blotting), the uncoupling from Gα_i/o_ in stably-transfected cells may be due to some aberration in structure and/or functionality when cultured long-term in the presence of antibiotic selection.

**Figure 6:**
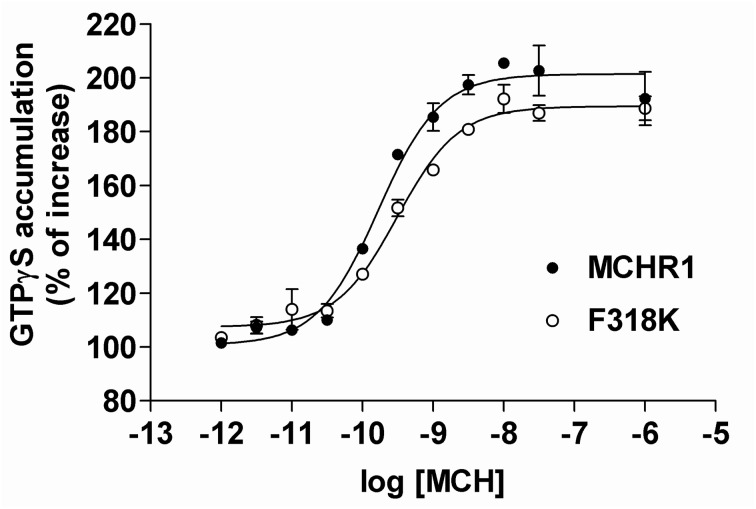
**MCH-induced [^35^S]GTPγ S binding to Flag-MCHR1 and F318K.** HEK293T cells were transfected with Flag-MCHR1 or F318K. After 48 h, the cells were harvested and the membrane fractions recovered. Membrane proteins (10 μ g) were subsequently incubated with 0.2 nM [^35^S]GTPγS and 0.001–1000 nM MCH in GTPγS binding buffer for 30 min at 30°C. The amounts of radioactivity bound to the membrane preparations are shown for Flag-MCHR1 (filled circles) and F318K (open circles). Results shown are representative of three-independent experiments.

Given the difficulties in measuring Gα_i/o_ protein activation using cAMP assays in stable clones, we employed an alternative technique. It is well-established that the key step in GPCR activation is the induction of guanine nucleotide exchange (GDP-GTP) on the G protein α-subunit. The nucleotide exchange process can be monitored by measuring the binding of non-hydrolyzable GTPγS analog, [^35^S]GTPγS. Because the Gα_i_ family of G proteins has a substantially higher basal rate of guanine nucleotide exchange than other G proteins, this assay is ostensibly a measure of GPCR-mediated activation of Gα_i/o_ proteins (Milligan, 2003). Indeed, the GTPγS binding assay has been widely used to measure the activation of Gα_i_ proteins by various GPCRs. Therefore, we examined both basal and MCH-stimulated [^35^S]GTPγS binding using the membrane fraction of HEK293T cells transiently expressing Flag-MCHR1 and the F318K mutant (Figure 6). In cells expressing Flag-MCHR1, MCH dose-dependently stimulated binding of GTPγS with an EC_50_ value of 0.23 ± 0.07 nM, while the value for F318K was 0.47 ± 0.25 nM (mean ± S.E.M. from three-independent experiments). The values for F318K were slightly higher, but a significant difference was not observed. The maximal amount of binding for Flag-MCHR1 with 1 μ M MCH was 208.3 ± 25.2% of basal, while that for F318K was 190.6 ± 20.3% of basal. Overall, there was no difference in the amount of GTPγS binding between cells expressing Flag-MCHR1 and the F318K mutant, in contrast to the clear influence of the F318K mutation on Gα_q_-mediated signaling. There was no significant difference in basal GTPγS binding (100% for Flag-MCHR1 vs. 97.6 ± 0.4% for F318K), suggesting that the F318K mutation does not affect constitutive receptor activation.

### Interpretation of the functional importance of F318K in MCHR1

**Figure 7:**
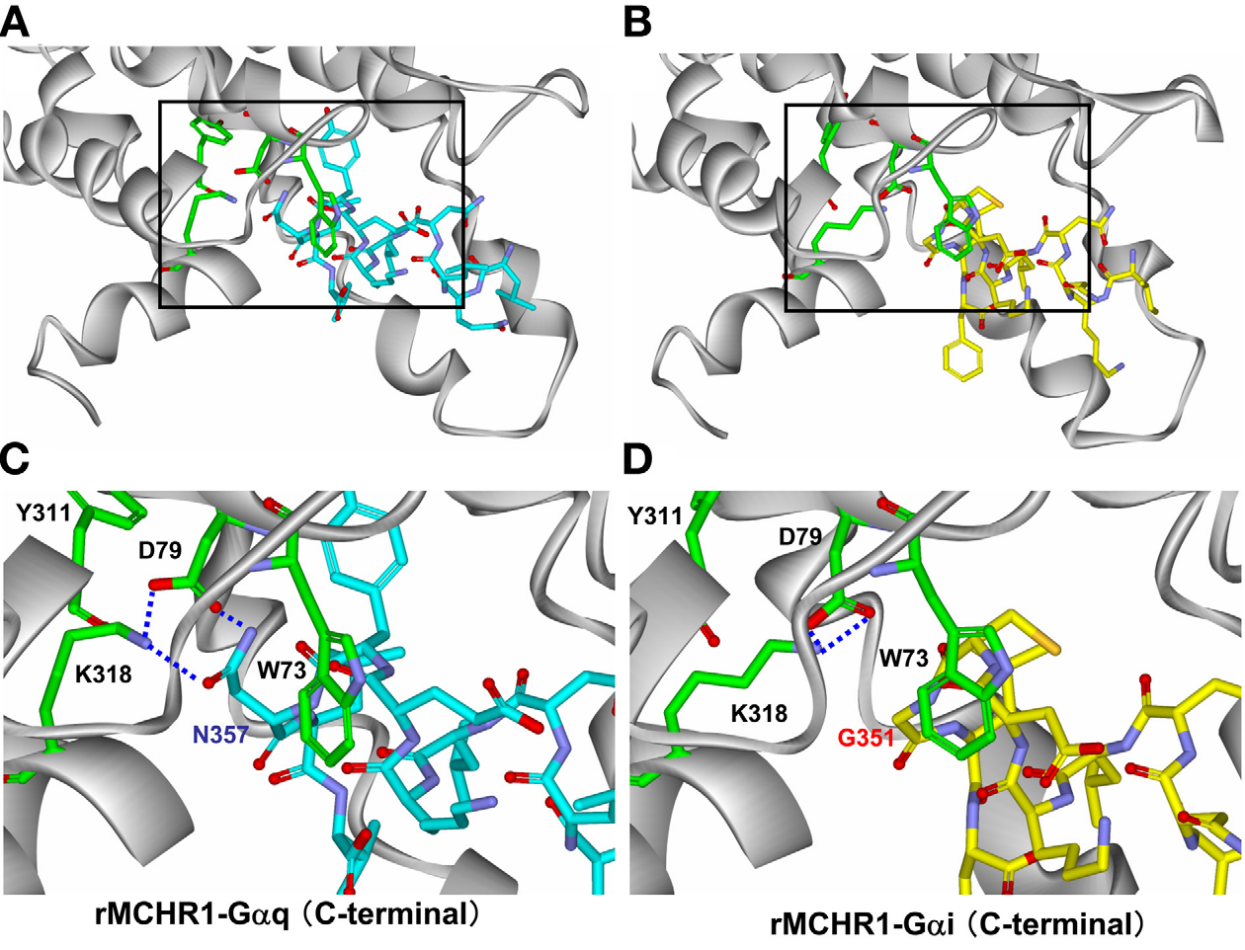
**Structural analysis of critical amino acids in a proposed model of rat MCHR1 mutant F318K with Gα_q_ or Gα_i_ proteins.** Homology model of rat MCHR1 mutant F318K was constructed using the crystal structure of rhodopsin E113Q mutant in complex with the C-terminal tail of transducin (PDB ID 2X72). Amino acid residues involved in coupling to Gα_q_ or Gα_i_ proteins in intracellular loop 1 (i1) and K318 in helix 8 are shown. **(A)** The model structure of MCHR1(F318K)-Gα_q_ C-terminal tail. The area demarcated by the square is magnified below in panel **(C)**. **(B)** The model structure of MCHR1(F318K)-Gα_i_ C-terminal tail. The area demarcated by the square is magnified below in panel **(D)**. **(C)** Positions of Lys318, Tyr311, Trp73, and Asp79 residues and their position relative to Asn357 of Gα_q_. This model depicts the hydrogen bond network between Lys318 and Asp79 in the receptor and Asn357 in Gα_q_ C-terminal tail. Note that interactions between Trp73 in i1 loop and Asn357 in the G protein C-terminal tail. Green, red, and blue areas indicate carbon, oxygen and nitrogen atoms, respectively, within the key residues of the receptor. Carbon atoms in the Gα_q_ protein are colored cyan. **(D)** Magnified view of Lys318, Tyr311, Trp73, and Asp79 residues in the receptor and their position relative to Gly351 of Gα_i_. Intramolecular hydrogen interactions predicted between Lys318 and Asp79 in the receptor are shown. Carbon atoms in the Gα_i_ protein are colored yellow.

To understand better how the signaling dynamics of F318K are related to its interaction with (and activation of) Gα_q_, we constructed a molecular model of F318K activation of G protein based on that for the active structure of rhodopsin in complex with a transducin peptide as a reference (Kleinau et al., 2010). Because MCHR1 belongs to the same GPCR subfamily as rhodopsin, the existing rhodopsin sequence alignment allowed us to construct a preliminary model of MCHR1 conformation. We refined this model to account for the specific properties of MCHR1 using software for protein model building and a molecular dynamics software package. Our model highlighted several putative amino acid contacts between the receptor and Gα_q_ protein, notably at intracellular loop 1 (i1) and helix 8 (Figure 7, left). Lys318 is predicted to interact efficiently with Asp79 (located in i1) *via* a distinct hydrogen-bonding pattern, and these two residues create an accessible intracellular interface for the Asn357 residue in the Gα_q_ protein. However, Asp79 in i1 also exists in human MCHR2, in which F313K had no effect on MCH potency in calcium signaling, as shown in Table 4. We therefore searched for alternative candidate residues that may be involved in the interaction with Asn357 by comparing sequences between rat MCHR1 and other GPCRs, including human MCHR2. The sequences we used are for GPCRs in which mutation at the position corresponding to Phe318 of MCHR1 did not produce increased signaling potency (Fritze et al., 2003; Delos Santos et al., 2006; Anavi-Goffer et al., 2007; Duvernay et al., 2009; Málaga-Diéguez et al., 2010; Kaye et al., 2011). By making multiple alignments of these sequences, we found several amino acid insertions in i1 that occurred only in rat MCHR1, and were absent in other GPCRs (Table 5). Notably, a sequence in i1 includes Trp73, which possesses a bulky, aromatic side chain. Hydrophobic interaction of Trp73 with Asn357 in the Gα_q_ C-terminal tail is predicted to stabilize the intracellular interaction between MCHR1 and Gα_q_. Conversely, the Gly351 residue in Gα_i_ has no side chain to interact with Lys318 (Figure 7, right), which may explain the lack of enhancing effect of the F318K mutation on Gα_i_-mediated signaling. Collectively, a predicted model of F318K combined with sequence alignments suggests that the key residues of the MCHR1-Gα_q_ interface are Trp73 and Asp79 in the receptor and Asn357 in the Gα_q_ protein. We speculate that the F318K mutation somehow enhances this interface, either by promoting heightened interaction or by facilitating G protein activation, possibly by changing the binding affinity (see section “Discussion”).

**Table 5:**
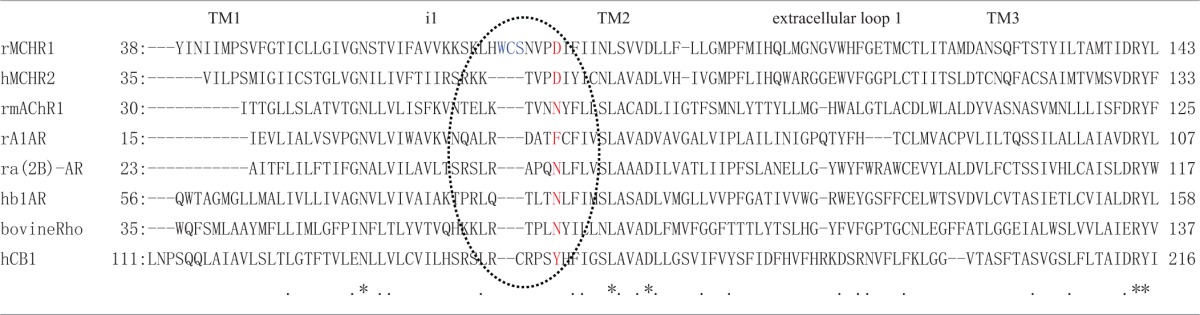
**Amino acid sequence alignment from the transmembrane 1 (TM1) to TM3 of rat MCHR1 and other GPCRs**.

Stars beneath sequence indicate sequence identity across the alignment, and single dot indicates conserved substitutions.

hMCHR2, human MCHR2; rmAChR1, rat muscarinic acetylcholine receptor M1; rA1AR, rat adenosine 1A receptor; ra(2B)-AR, rat α2B adrenergic receptor; hb1AR, human β1 adrenergic receptor; bovineRho, bovine rhodopsin; hCB1, human cannobinoid receptor 1.

## Discussion

Recent studies have underscored that, the Phe residue is a key position in the conserved NPxxY(x)_5,6_F motif, which connects TM7 and helix 8 (Palczewski et al., 2000; Fritze et al., 2003). Indeed, of 180 class A GPCRs, this Phe residue is the most highly conserved in 135 of these receptors (Okuno et al., 2005). Mutation of the conserved Phe to Ala in rhodopsin and the M1 muscarinic acetylcholine receptor considerably reduced the potency in Gα_t_- and Gα_q_-mediated signaling, respectively (Fritze et al., 2003; Kaye et al., 2011). Furthermore, Phe-to-Ala substitution in this motif dramatically reduced cell surface expression of α2B-AR, resulting in extensive ER arrest (Duvernay et al., 2009). These data suggest that the highly hydrophobic Phe residue in the conserved NPxxY(x)_5,6_F motif of many GPCRs is necessary for the maintenance of proper receptor conformation, and is often involved in receptor export from the ER. In the present study, we showed that Phe-to-Ala substitution (F318A) in rat MCHR1 did not significantly affect either the level of cell surface expression or MCH-induced calcium mobilization. However, by analyzing a series of substitution mutants, Phe-to-Lys substitution led to the most increased MCH potency in stimulating calcium mobilization, a pathway largely mediated *via* Gα_q_. In contrast, F318K had no significant effect in the GTPγS-binding assay that measures receptor interaction with Gα_i_ protein. These findings are surprising considering that they contradict findings in Phe substitutions of other GPCRs. Therefore, our study provides new insight into the role of the NPxxY(x)_5,6_F motif Phe residue in term of G protein activation. Although the consensus is that this residue is a key determinant of receptor structure and function, its effect may be dependent on the specific sequence of the receptor and the presence or absence of other residues that make up the receptor-G protein binding/activation interface.

We showed that MCH was the most potent in stimulating calcium mobilization in cells expressing F318K among our mutants. To date, many mutations or truncations in MCHR1 have been designed to study the roles of certain residues and sequences in the diverse functionality of the receptor (Macdonald et al., 2000; Saito et al., 2003, 2004, 2005; Tetsuka et al., 2004; Fan et al., 2005). Some caused a slight or moderate decrease in calcium mobilization (Saito et al., 2003, 2004) and the others resulted in more severe impairment or loss of function (Macdonald et al., 2000; Tetsuka et al., 2004; Fan et al., 2005; Saito et al., 2005; Aizaki et al., 2009). F318K is, therefore, the first mutation characterized by an active variant with more efficient signaling properties. We conclude that substitution of Phe318 with the moderately positively charged Lys is most responsible, at least in part, for producing increased MCH potency in stimulating calcium mobilization *via* MCHR1. Replacement with another positively charged amino acid, His, at the 318 position had no effect for signaling enhancement. Furthermore, the highly positively charged Arg could not induce equivalent effects to F318K (Figure 2, Table 3). These findings suggest that unique and strict physicochemical characteristics, including the extent of positive charge, polarity and the size of the side chain, may be required for this phenomenon to occur.

GPCRs that bind promiscuously to several Gα protein subtypes are useful tools for clarifying the determinants of G protein selectivity. To date, most studies of rhodopsin family GPCRs have emphasized the role of membrane-proximal regions in the i2 and i3 loops and/or the cytoplasmic loop of the receptor (Anavi-Goffer et al., 2007; Kunieda et al., 2007; Kleinau et al., 2010). Our present data suggest that MCHR1 couples to both Gα_i_ and Gα_q_ proteins *via* overlapping intracellular regions but that Lys mutation of the 318 position in the NPxxY(x)_5,6_F motif appears to affect only the coupling of the receptor to Gα_q_. Phe318 is located in the juxtamembrane helix 8 in the C-terminal tail. The role of helix 8 is believed to be in regulating G protein activation by GPCRs, yet few studies have established the pivotal role of helix 8 in G protein selectivity. The position in cannabinoid receptor 1 that corresponds to the MCHR1 Phe318 residue is not a Phe but is a hydrophobic residue (Leu404), and the L404I mutation led to impaired activation of Gα_i3_ but not Gα_oA_ among the Gα_i/o_ protein subgroup (Anavi-Goffer et al., 2007). Furthermore, the positively charged Arg687 in helix 8 of the thyrotropin receptor is important for selective interaction with Gα_q_ (Kleinau et al., 2010). In helix 8 of MCHR1, three basic amino acid residues (Arg319, Lys320, and Arg321) are juxtaposed with Phe318. This basic region in helix 8 seems not to be involved in Gα protein coupling specificity, since alteration of the positive charge of these residues (R319Q/K320Q/R321Q) caused an equally strong inhibition of both Gα_q_ and Gα_i_ (Tetsuka et al., 2004). Taken together, we might conclude that each amino acid residue in helix 8 of MCHR1 may have different roles in determining receptor activity and G protein selectivity. Our clarification here of the significance of the Phe position will hopefully allow additional understanding of dual G protein coupling in other GPCRs.

Upon stimulation, GPCRs undergo a conformational change that results in G protein activation. Light-induced conformational changes in rhodopsin were elucidated by a series of biophysical studies. Evidence indicates that an important hydrophobic pairing between Tyr306 and Phe313 in the NPxxY(x)_5,6_F motif stabilizes the ground state of rhodopsin (Palczewski et al., 2000; Nygaard et al., 2009). Comparative molecular dynamics simulations of MCHR1 have also suggested that the release of intramolecular interactions between the Tyr and Phe residues in the motif is an important step in transitioning from an inactive to an active form (Vitale et al., 2004). We initially hypothesized that the Tyr311-Phe318-based switch in MCHR1, which is the homolog of a crucial relay point in rhodopsin, would be modified by substituting Phe with Lys, with disruption of an aromatic stacking interaction in the resultant ternary complex somehow affecting the efficacy of signaling. However, the activated F318K structure bound with Gα_q_ protein (Figure 7) has suggested that an additional and distinct interface may exist between Phe318 and Asp79 in the i1 loop. Further analysis with multiple alignment data for other GPCRs showed that close proximity of Phe318, Asp79, and Asn357 is insufficient to induce such signaling changes and that Trp73 in the i1 loop may be important in stabilizing/maintaining the efficient binding/activation of the Gα_q_ protein by the activated receptor. A series of combinational mutations involving F318K and several amino acid residues in the i1 loop (Trp, Cys, and Ser, as described in Table 5) may clarify the importance and role of each residue. A precise three-dimensional crystal structure of the MCHR1-G protein complex would also facilitate our understanding, but our present model helps to understand how a single amino acid substitution, F318K, can alter the intra-/inter-molecular interactions of the receptor-G protein complex to modulate G protein signaling *via* this receptor. At this time, we cannot exclude the possibility that F318K has an increased binding affinity, although the EC_50_ value of MCH in the GTPγS-binding assay showed no change between Flag-MCHR1 and F318K. The concept of modes in agonism of GPCRs has recently been expanding (Smith et al., 2011), implying that receptors can convey different signals through different pathways with varying degrees of potency or efficacy depending on the ligand used. Therefore, it may be possible that the functional selectivity observed in F318K results from enhancement of the binding affinity that favors Gα_q_-binding but not Gα_i_-binding. Overall, based on our data, we favor an interpretation that F318 is involved in receptor dynamics.

In conclusion, by analyzing a series of mutants, we report for the first time that F318K, a point mutation in the NPxxY(x)_5,6_F motif of MCHR1, most efficiently enhances the potency of MCH in stimulating calcium mobilization *via* MCHR1 without showing any increase in cell surface expression. We speculate that Phe318 might be involved in the interface between the receptor and G protein, and may regulate which G proteins the receptor can bind and activate. Because there are a limited number of GPCR mutant that significantly enhanced the signaling (Reinscheid et al., 2005; Kato et al., 2008), F318K may provide useful clues in understanding the process of intrinsic receptor dynamics. However, further research is required to demonstrate whether the role of Phe318 described here is of physiological and functional significance.

### Conflict of interest statement

The authors declare that the research was conducted in the absence of any commercial or financial relationships that could be construed as a potential conflict of interest.

## Abbreviations

ECL: enhanced chemiluminescence
ER: endoplasmic reticulum
ERK: extracellular signal-regulated kinase
FBS: fetal bovine serum
GPCR: G-protein-coupled receptor
HEK293T: human embryonic kidney 293
helix 8: eighth cytoplasmic helix
i1: intracellular loop 1
i2: intracellular loop 2
i3: intracellular loop 3
MCH: melanin-concentrating hormone
MCHR1: MCHR1 receptor
MCHR2: MCHR2 receptor
PTX: pertussis-toxin
TM: transmembrane.
